# Rice *OsGL1-6* Is Involved in Leaf Cuticular Wax Accumulation and Drought Resistance

**DOI:** 10.1371/journal.pone.0065139

**Published:** 2013-05-31

**Authors:** Lingyan Zhou, Erdong Ni, Jiawei Yang, Hai Zhou, Hong Liang, Jing Li, Dagang Jiang, Zhonghua Wang, Zhenlan Liu, Chuxiong Zhuang

**Affiliations:** 1 Laboratory Center of Basic Biology and Biotechnology, Education Department of Guangdong Province, College of Life Sciences, Zhongkai University of Agriculture and Engineering, Guangzhou, Guangdong, People’s Republic of China; 2 State Key Laboratory for Conservation and Utilization of Subtropical Agro-bioresources, College of Life Sciences, South China Agricultural University, Guangzhou, Guangdong, People’s Republic of China; 3 College of Agronomy, Northwest A&F University, Yangling, Shanxi, People’s Republic of China; BASF Cropdesign, Belgium

## Abstract

Cuticular wax is a class of organic compounds that comprises the outermost layer of plant surfaces. Plant cuticular wax, the last barrier of self-defense, plays an important role in plant growth and development. The *OsGL1-6* gene, a member of the fatty aldehyde decarbonylase gene family, is highly homologous to *Arabidopsis CER1*, which is involved in cuticular wax biosynthesis. However, whether *OsGL1-6* participates in cuticular wax biosynthesis remains unknown. In this study, an *OsGL1-6* antisense-RNA vector driven by its own promoter was constructed and introduced into the rice variety Zhonghua11 by *Agrobacterium*-mediated transformation to obtain several independent transgenic plants with decreased *OsGL1-6* expression. These *OsGL1-6* antisense-RNA transgenic plants showed droopy leaves at the booting stage, significantly decreased leaf cuticular wax deposition, thinner cuticle membrane, increased chlorophyll leaching and water loss rates, and enhanced drought sensitivity. The *OsGL1-6* gene was constitutively expressed in all examined organs and was very highly expressed in leaf epidermal cells and vascular bundles. The transient expression of *OsGL1-6-GFP* fusion indicated that OsGL1-6 is localized in the endoplasmic reticulum. Qualitative and quantitative analysis of the wax composition using gas chromatography-mass spectrometry revealed a significantly reduced total cuticular wax load on the leaf blades of the *OsGL1-6* antisense-RNA transgenic plants as well as markedly decreased alkane and aldehyde contents. Their primary alcohol contents increased significantly compared with those in the wild type plants, suggesting that *OsGL1-6* is associated with the decarbonylation pathways in wax biosynthesis. We propose that *OsGL1-6* is involved in the accumulation of leaf cuticular wax and directly impacts drought resistance in rice.

## Introduction

The cuticle is a continuous hydrophobic lipid layer structure that covers the exposed ground parts of terrestrial plants and forms a protective barrier against the external environment. The cuticle is synthesized by the epidermal cells and is composed of cutin polymer matrix and waxes [1], [2]. Cuticular waxes comprise the primary structure of the cuticle and play the following important roles: limiting non-stomatal water loss [3]; repelling bacterial and fungal pathogens and herbivorous insects [4]; mediating the interaction with other organisms, such as bacteria, fungi, and insects [5]–[7]; and preventing UV radiation and frost damage [2], [8].

The cuticular wax of all plants consists of derivatives of very long-chain fatty acid (VLCFAs) including alkanes, aldehydes, ketones, primary and secondary alcohols, and esters [9], [10]. The biosynthesis of cuticular wax is accomplished by two steps: the fatty acid elongase-mediated extension of the C16 and C18 fatty acids to VLCFA chains and the conversion of each VLCFA to wax components by the decarbonylation and acyl reduction pathways in the endoplasmic reticulum (ER). The acyl reduction pathway mediates the production of primary alcohols and wax esters, whereas the decarbonylation process produces aldehydes, secondary alcohols, alkanes, and ketones [9], [10].

The genes associated with wax synthesis have been successfully isolated and identified from maize and *Arabidopsis* using genetic analysis. Among these genes, *FAE1*, *FDH*, *KCS1*, *PAS2*, *CER6*, *CER10*, *GL8A* and *GL8B* are involved in the synthesis of very long-chain fatty acid wax precursors [11]–[19]. Genes including *CER4*, *WSD1* and *MAH1* are involved in the synthesis of wax components [20]–[22], of which, *CER4* and *WSD1* participate in the acyl reduction pathway to catalyze the production of primary alcohol and wax ester, respectively [20], [21]. *MAH1* participates in the decarbonylation pathway to catalyze the conversion of alkanes into secondary alcohols and ketones [22]. *CER5* and *LTPG* are involved in wax secretion [23]–[25]. Genes such as *WIN1/SHN* encode regulatory proteins [26], [27]. All known VLCFA elongases and wax biosynthesis enzymes are located in the ER, and the cuticular lipids synthesized in the ER must be transported to the cuticle, where they apparently self-assemble into the cuticle proper [10]. *CER5* in *Arabidopsis* is the first characterized gene that encodes the plasma membrane-localized ABC transporter required for the transport of wax components from the epidermal cells to the cuticle [23].

*LTPG* in *Arabidopsis* encodes a glycosylphosphatidylinositol-anchored lipid transfer protein that is localized in the plasma membrane of stem epidermal cells. *LTPG* has the capacity to bind to the lipid, which is required for the export of lipids to the plant surface, suggesting that *LTPG* may function as a component of the cuticular lipid export machinery [24], [25]. *WIN1/SHN* encodes an APETALA2/EREBP-type transcription factor [26], [27]. Overexpression of *WIN1/SHN1* increases wax production and enhances the drought tolerance in *Arabidopsis* [28]. *WXP1* is a putative AP2 domain-containing transcription factor gene from the model legume *Medicago truncatula* and activates wax production in the acyl reduction pathway. Overexpression of *WXP1* also leads to increased cuticular wax loading on the leaf surfaces, reduced water loss, and enhanced drought tolerance in transgenic alfalfa [29].

Many wax-defective mutants were obtained in *Arabidopsis*, maize and other plants that either showed a reduced wax accumulation or modified wax composition and were often characterized by their smooth or light green phenotype. *CER1* and *CER3/WAX2/YRE/FLP* in *Arabidopsis* and *GL1* in maize have been isolated and characterized using wax-defective mutants. The *cer1* mutants in *Arabidopsis* display glossy green stems and fruits. Biochemical studies have shown that the *cer1* mutants exhibited a significant reduction of alkanes, secondary alcohols, and ketones as well as an increased aldehyde content. CER1 was predicted to encode an aldehyde decarboxylase, a key wax biosynthetic enzyme that catalyzes the conversion of aldehyde to alkane [9], [30]–[34]. The *cer1* mutants were conditionally male sterile, suggesting that the *CER1* gene has an essential function in pollen development [31]. Overexpression of *CER1* dramatically increased alkane production and resulted in an increased wax load, reduced cuticle permeability, and enhanced plant stress resistance [33]. A screen for CER1 physical interaction partners revealed CER1 interacts with the wax-associated protein CER3 and ER-localized cytochrome b5 isoforms (CYTB5s) [34].

The *cer3* (*cer3/wax2/yre/flp1*) mutants showed glossy stems as well as altered cuticle membrane and cuticular waxes. Biochemical studies have shown that the aldehyde, hydrocarbon, ketone, and secondary alcohol contents were significantly reduced in the mutants [35]–[37], suggesting that *CER3/WAX2/YRE/FLP* might encode an aldehyde-producing enzyme that catalyzes the conversion of acyl-CoA to an intermediate aldehyde [36]. The *gl1* mutants showed waxless seedling leaves and altered cuticle membrane and cuticular waxes similar to those observed in the *wax2* mutant. Levels of the aldehydes and alcohols in the *gl1* mutant were markedly reduced, indicating that *GL1* is essential for the elongation process during the synthesis of cuticular wax [38].

Until now, three numbers of the fatty aldehyde decarbonylase gene family were characterized in rice. *Wda1*, the first characterized gene, is strongly expressed in the epidermal cells of the anthers. The *wda1* mutant demonstrated absent epicuticular wax crystals in the outer layer of the anther and severely retarded microspore development. Contents of the fatty acids, alkanes, alkenes, and primary alcohols in the *wda1* mutant were severely reduced, indicating that *Wda1* may be involved in the general processes of VLCFA biosynthesis [39]. The *osgl1-1* (also known as *wsl2*) mutant showed a decreased cuticular wax deposition and enhanced drought sensitivity [40], [41]. A wax composition analysis revealed a substantially reduced quantity of C22–C32 fatty acids in the *wsl2* mutant, suggesting that *WSL2* may be involved in the VLCFA elongation [41]. The *osgl1-2* mutant showed reduced cuticular wax synthesis and increased sensitivity to drought stress, *while OsGL1-2* overexpression resulted in increased amounts of cuticular waxes and enhanced drought tolerance [42]. Hence, members of the fatty aldehyde decarbonylase gene family play important roles in cuticular wax synthesis and drought resistance.

A systematic sequence analysis revealed a total of 11 *GL1* homologous genes in rice, designated *OsGL1-1* to *OsGL1-11* [42]. However, only three genes, i.e. *Wda1* (also called *OsGL1-5*), *OsGL1-1* (*WSL2*), and *OsGL1-2*, were characterized. The mutations in the three genes confer different phenotypic changes on the cuticular wax compositions, implying that they may perform different roles in cuticular wax biosynthesis. To better understand the molecular mechanism of cuticular wax biosynthesis in rice, more putative genes that are deduced to participate in the process need to be characterized.

In this study, we conducted a functional analysis of *OsGL1-6* in rice growth and development by generating *OsGL1-6* antisense-RNA transgenic plants. *OsGL1-6* is homologous to *CER1* in *Arabidopsis* and *Wda1* in rice, universally expressed in vegetative and reproductive organs, and especially highly expressed in leaf epidermal cells and vascular bundles. The results of a phenotypic characterization and drought sensitivity experiment of *OsGL1-6* antisense-RNA transgenic plants indicated that *OsGL1-6* is involved in cuticular wax accumulation and drought resistance.

## Materials and Methods

### Plants and Other Experimental Materials

The rice variety Zhonghua11 (*Oryza sativa* L. ssp. *japonica* cv. Zhonghua11) was used for all of the experiments in this study. The wild type (WT) and transgenic plants used in the drought stress experiments were hydroponically cultured as reported previously [43], and all rice plants were grown in a greenhouse under normal growth conditions. *Escherichia coli* strain DH10B and *Agrobacterium tumefaciens* strain EHA105 were used for the cloning and transformation experiments. pCAMBIA1380 was used as the binary vector for *Agrobacterium*-mediated transformation.

### Generating *OsGL1-6* Antisense-RNA Transgenic Plants

The *OsGL1-6* antisense transgene driven by the cognate promoters of the *OsGL1-6* was constructed following the protocol described by Li et al. [44]. A 624-bp fragment of the *OsGL1-6* cDNA was amplified using reverse transcription polymerase chain reaction (RT-PCR) from Zhonghua11 with the gene-specific *OsGL1-6*Af and *OsGL1-6*Ar primers (Table S1), and the PCR product was inversely inserted into the multiple cloning site of pCAMBIA1380. A 1946-bp genomic fragment located upstream of the annotated ATG start codon of the *OsGL1-6* was isolated from Zhonghua11 by using *OsGL1-6*Pf and *OsGL1-6*Pr primers (Table S1) and was then inserted upstream of the antisense fragment. The construct was then transformed into *A. tumefaciens* strain EHA105, which was used to transform rice calli generated from mature seeds of Zhonghua11 according to a previously described protocol [45].

### RT-PCR Analysis

Total RNA from different Zhonghua11 tissues and transgenic plant seedlings was extracted using TRIzol reagent (Invitrogen, Carlsbad, CA, USA) according to the manufacturer’s instructions. Prior to the RT-PCR procedure, total RNA was treated with RNase-free DNase I (Promega, Carlsbad, CA, USA) to degrade any residual genomic DNA. The first-strand cDNAs were synthesized at 42°C for 1 h in a 20-µL reaction containing 1.0 µg of total RNAs, 4.0 µL of 5×reaction buffer, 1.0 µL of oligo d(T)_15_ (50 mM), 2.0 µL of dNTP mix (10 mM each), 1.0 µL of Ribonuclease Inhibitor (40 U/µL; Takara, Dalian, China), and 1 µL of avian myeloblastosis virus reverse transcriptase (5 U/µL; Takara, Dalian, China). PCR was performed in a 50-µL reaction containing 1 µL of the first-strand cDNA template, 40 µL of ddH_2_O, 5 µL of 10×PCR buffer, 0.4 µL of dNTP mix (10 mM each), 1 µL of forward primer (10 µM), 1 µL of reverse primer (10 µM), and 0.8 µL of DNA polymerase (2.5 U/µL; Takara, Dalian, China). PCR was performed using the following cycling profile: 94°C for 5 min, 28 cycles at 94°C for 30 s, 56°C for 30 s, 72°C for 45 s, and then 72°C for 10 min. Four pairs of primers (*OsGL1-6*-RTf and *OsGL1-6*-RTr, *Wda1*-RTf and *Wda1*-RTr, *OsGL1-1*-RTf and *OsGL1-1*-RTr, and *OsGL1-2*-RTf and *OsGL1-2*-RTr) were used for the semi-quantitative RT-PCR (Table S1). The *OsAct1* gene (rice *Actin1*, NM_001057621, Os03g0718100) was used as an internal control for the RT-PCR and was amplified by primer pair *OsAct1*f and *OsAct1*r (Table S1).

### Histochemical β-glucuronidase (GUS) Analysis

A modified pCAMBIA1300 vector containing the CaMV35S promoter, *GUS* gene, and *NOS* terminator inserted into the multiple cloning sites was used for the GUS fusion construction [46]. A 1,946-bp genomic fragment located upstream of the annotated ATG start codon of *OsGL1-6* was isolated from Zhonghua11 using the primer pair *OsGL1-6*Gf and *OsGL1-6*Gr (Table S1) and then substituted for the CaMV35S promoter in the above construct. The *GUS* fusion construct was then transformed into Zhonghua11 using *Agrobacterium*-mediated transformation [45]. For the GUS staining, the tissues were fixed for 45 min in a 50 mM sodium phosphate buffer, pH 7.0, containing 1% (v/v) formaldehyde and 0.5% (v/v) Triton X-100 after being vacuumed for 10 min. The fixed tissues were rinsed and vacuum-infiltrated three times (5 min each) in 50 mM sodium phosphate buffer, pH 7.0, containing 0.5% (v/v) Triton X-100, 1 mM potassium ferricyanide and 1 mM potassium ferrocyanide. The above tissues were subsequently stained in a 1 mM sodium phosphate buffer, pH 7.0, containing 0.5% (v/v) Triton X-100, 0.25 mg/mL X-Gluc, and 1 mM potassium ferrocyanide at 37°C overnight after being vacuumed for 10 min. The samples were then rinsed and stored in 70% (v/v) ethanol and observed under a microscope (Nikon).

### Subcellular Localization

The CaMV35S promoter, *eGFP/CFP* fragments, and *NOS* terminator were successively cloned into pUC18 to construct the transient expression vectors. The intact open reading frame (ORF) of *OsGL1-6* was amplified using the primer pair *OsGL1-6*-eGFPf, *OsGL1-6*-eGFPr (Table S1) and then subcloned into the transient expression vector between the CaMV35S promoter and the *eGFP* gene, generating in-frame with the N-terminus of *eGFP* being driven by the CaMV35S promoter. The *CFP-KDEL* fusion transient expression vector was constructed by subcloning of the ER retention signal *KDEL* to the 3′ end of the *CFP* gene [47], and sequences of the used primers were listed in Table S1. Protoplasts from the leaf sheaths of the 14-day rice seedlings were prepared and transient expression was analyzed following a described protocol [48], [49].

### Western Blot Analysis

The polyclonal antibody of OsGL1-6 for the Western blot analysis was made in our laboratory. The intact ORF of *OsGL1-6* was amplified using primer pair *OsGL1-6*-pETf, *OsGL1-6*-pETr (Table S1) and then cloned into pET-23d. The resulting recombinant vector was introduced into *E. coli* strain BL21 (DE3) and induced to express the OsGL1-6 fusion protein using isopropylthio-β-galactoside. The polyclonal antibodies were obtained using OsGL1-6 fusion protein as the antigen in immune rabbits. Seedlings (0.3 g) from the WT and transgenic plants were ground into a homogenate in an extraction buffer, pH 7.5, containing 0.1 M Tris-HCl and 25 mM ethylenediaminetetraacetic acid. The homogenate was centrifuged at 12,000×*g* for 10 min at 4°C, and the upper aqueous solution containing the proteins was collected. The immunoblot analysis was performed as described previously by Li et al. [44]. Heat shock protein 90 (BGI, Shenzhen, China) was used for reference [50].

### Drought Sensitivity and Cuticular Permeability Experiment

The seeds of the WT and transgenic plants were germinated and cultured on half-strength Murashige and Skoog's medium under a 12 h light/12 h dark schedule at 26°C for 4 d. The seedlings were then transferred out and hydroponically cultured for 10 d in a greenhouse. Drought treatment was subsequently performed in the above seedlings in the air for 10 h. The plants were re-watered for 7 d, and plant survival rates were calculated.

The chlorophyll leaching experiment was performed as previously described [40]. Briefly, the second leaves from the top were sampled from each tiller at the plant booting stage, and the leaves were then cut into segments and immersed in 30 mL of 80% (v/v) ethanol with gentle agitation in the dark. At 10, 20, 30, 40, 50, 60, 90, 120, and 150 min, 3-mL aliquots were taken for chlorophyll quantification. These samples were poured back to the same tube after the measurements. The chlorophyll concentration was quantified using a spectrophotometer at wavelengths of 664 and 647 nm.

For the water-loss measurements, the second leaves from the top were detached from each tiller at the plant booting stage and soaked in deionized water for >2 h in the dark. The detached leaves were subsequently removed from soaking, blotted gently with a napkin to remove excess water, and weighed at 0.5, 1, 1.5, 2, 2.5 and 3 h as described by Chen et al. [35]. Measurements were performed in a very low-light environment. The percentage of fresh weight loss was calculated based on the initial sample weight.

### Scanning Electron Microscopic Analysis

The tips of the booting-stage flag leaves were fixed at 4°C in a 0.1 M sodium phosphate buffer, pH 6.8, containing 2.5% (v/v) glutaraldehyde for 24 h. The samples were rinsed three times (10 min each) in a 0.1 M sodium phosphate buffer, pH 6.8, dehydrated first with an ethanol series (10 min each) of 50% to 100% (v/v) and then twice (15 min each) with 100% (v/v) acetone, and then exchanged three times (15 min each) with isoamyl acetate. The samples were processed for critical point drying in liquid CO_2_ (Bal-Tec), gold-coated (10-nm-thick), and examined in an XL-30-ESEM (FEI) with an accelerating voltage of 20 kV.

### Transmission Electron Microscopic Analysis

The tips of the booting-phase flag leaves were fixed for 24 h in sodium phosphate buffer, pH 7.2, containing 4% (v/v) glutaraldehyde and 3% (w/v) paraformaldehyde. The samples were rinsed three times (20 min each) in sodium phosphate buffer, pH 7.2 and then post-fixed for 1.5 h in sodium phosphate buffer, pH 7.2, containing 1% (v/v) osmium tetroxide. The samples were subsequently dehydrated with an ethanol series (10 min each) of 50% to 100% (v/v) and twice (15 min each) with 100% acetone. After dehydration, the specimens were infiltrated and embedded in Epon-812 Resin. Ultrathin sections (50–70 nm) were prepared using a Leica UCT ultramicrotome. The sections were mounted on copper grids and stained for 10 min with 4% (w/v) uranyl acetate and lead citrate and examined at 60–80 kV using a TECNAI G2 12 transmission electron microscope (FEI).

### Wax Extraction and Composition Analysis

The total wax content was determined using the weight method. In brief, 2 g of the booting-stage flag leaves was immersed in 30 mL of chloroform at 60°C for 30 s, and the wax content was then determined by analytical balance after complete chloroform evaporation.

The leaf wax composition was analyzed as previously described [39]. Briefly, five 10-cm booting-stage flag leaf blades were immersed in 30 mL of chloroform at 60°C for 30 s; 20 µg of tetracosane was used as an internal standard. The solvent was evaporated under a nitrogen stream. The samples were transferred to gas chromatography (GC) vials and converted to the trimethylsilyl derivatives in 10 µL of pyridine and 10 µL of bis-(*N*, *N*-trimethylsilyl)-tri-fluoroacetamide (BSTFA) for 40 min at 70°C. After cooling, the BSTFA and pyridine were removed under a nitrogen stream and their derivatives were dissolved in 100 µL of chloroform for gas chromatography-mass spectrometry (GC-MS) analysis. The wax composition was determined using a GC (HP1-MS) column and mass spectrometric detector (5973N, Agilent). The wax loads were estimated by quantifying the major peak areas compared with the internal standard area. The GC was conducted with an on-column injection at an initial oven temperature of 50°C for 2 min, followed by an increase of 40°C/min to 200°C, 2 min at 200°C, an increase of 3°C/min to 320°C, 30 min at 320°C, and helium carrier gas at 2 mL/min.

## Results

### Prediction of *OsGL1-6* Function

**Figure 1:**
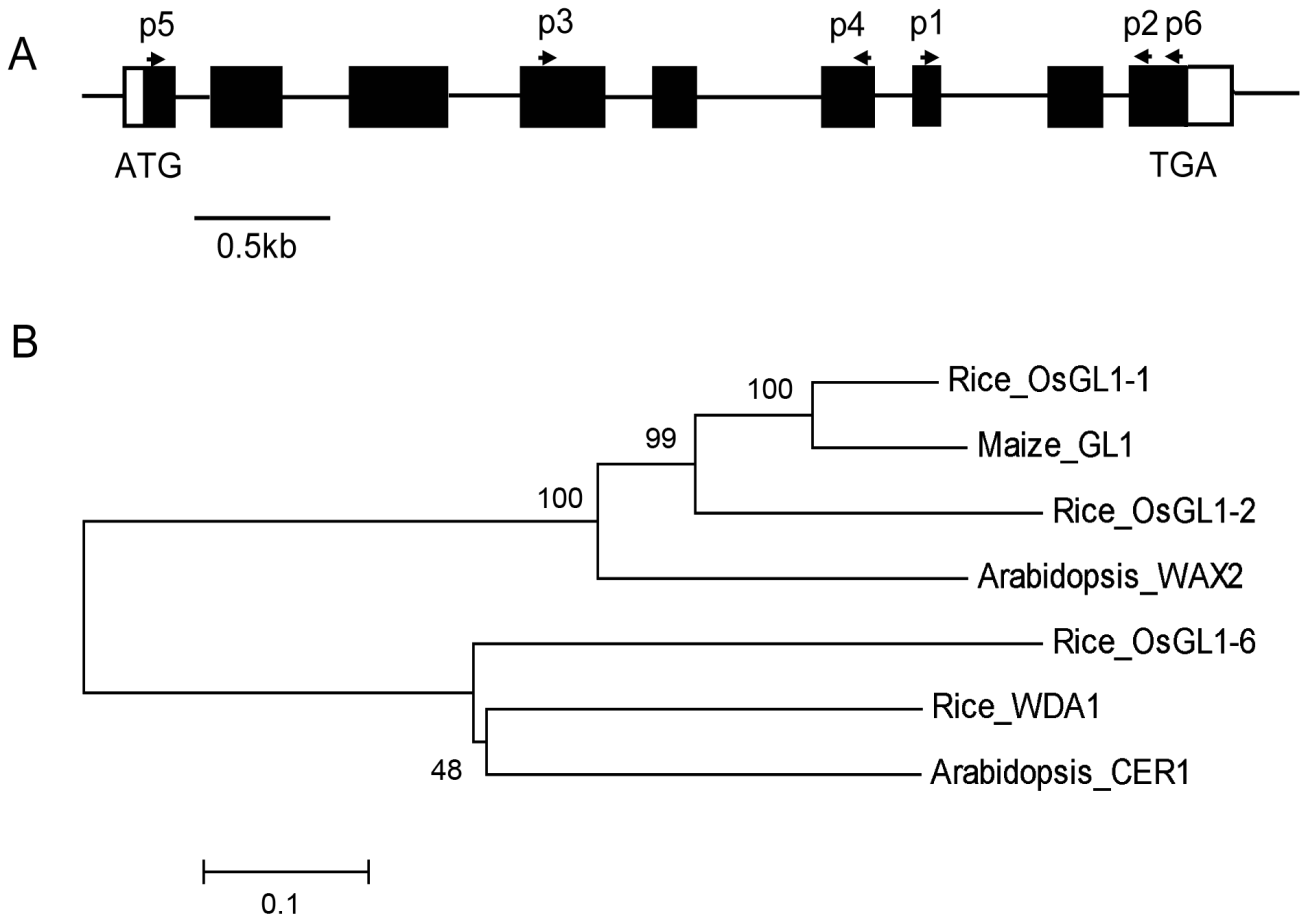
Scheme of the *OsGL1-6* gene and phylogenic analysis of OsGL1-6-related proteins. (A) Genomic organization of *OsGL1-6* gene. The closed black boxes indicate exons, connecting lines indicate introns, and closed white boxes indicate the 5′ and 3′ untranslated region. The ATG start codon and TGA stop codon are also indicated. p1 and p2 are the primers used in the semi-quantitative reverse transcription polymerase chain reaction analysis of the *OsGL1-6* transcript. p3 and p4 are the primers used to amplify the antisense fragment. p5 and p6 are the primers used to amplify the intact ORF. (B) Phylogenic analysis of proteins homologous to OsGL1-6. The coding region sequences were aligned using Clustal W [51] and subjected to neighbor-joining analysis as implemented in MEGA5.0 [52]. Bar = 0.5 kb.

According to the DNA sequence in the NCBI database, *OsGL1-6* is present on chromosome 2 of the rice genome and contains nine exons and eight introns (Figure 1A). The full length of *OsGL1-6* cDNA (NM_001055025) is 2,218 bp. The ORF of *OsGL1-6* is 1,908 bp, and it encodes a protein with 635 amino acids that has a molecular mass of 71.6 kD and an isoelectric point of 8.64 and that belongs to the fatty aldehyde decarbonylase superfamily. Conserved domain analysis using the Conserved Domain Database program (http://www.ncbi.nlm.nih.gov/Structure/cdd/wrpsb.cgi) showed that the N-terminus of OsGL1-6 contains a conserved FA_hydroxylase domain of the fatty acid hydroxylase superfamily. The members of this family are membrane proteins and are involved in the biosynthesis of plant cuticular wax [31]. The prediction of a signal peptide and subcellular localization using SignalP3.0 (http://www.cbs.dtu.dk/services/SignalP/) and PSORT (http://psort.nibb.ac.jp/form.html) showed no signal peptide at the N-terminus of OsGL1-6. However, OsGL1-6 has an N-terminal transmembrane domain and is potentially localized in the ER, peroxisome, and plasma membrane. The OsGL1-6 protein contains one His-rich motif, HX_3_HH, starting at the 147^th^ amino acid as well as two HX_2_HH motifs beginning at 160^th^ and 249^th^ amino acids. This domain forms a bivalent iron ion-binding site that is necessary for the catalytic activities of integrated membrane proteins such as sterol desaturase, acyl desaturase, and alkyl-hydroxylase [31].

OsGL1-6 showed high sequence similarities with the identified numbers of the fatty aldehyde decarbonylase superfamily. For example, OsGL1-6 has 53% sequence identity with CER1 and 33% homology with CER3/WAX2/YRE/FLP in *Arabidopsis*, 53% with Wda1, 33% with OsGL1-1, and 30% with OsGL1-2 in rice, and 33% with GL1 in maize.

Phylogenic analysis of the predicted protein sequences for the six characterized genes with high similarities to OsGL1-6 showed that they can be grouped into two clades (Figure 1B). OsGL1-6 groups with WDA1 and CER1, whereas OsGL1-1, OsGL1-2, maize GL1, and WAX2 form the other group. *Wda1* is involved in wax production in rice anther walls [39]. *CER1* functions in the biosynthesis of stem wax and, more specifically, in very-long-chain alkane biosynthesis [31]–[34]. The phylogenic analysis results suggested that *OsGL1-6* is likely involved in wax biosynthesis.

### *OsGL1-6* Expression within Epidermal Cells and Vascular Bundles

**Figure 2:**
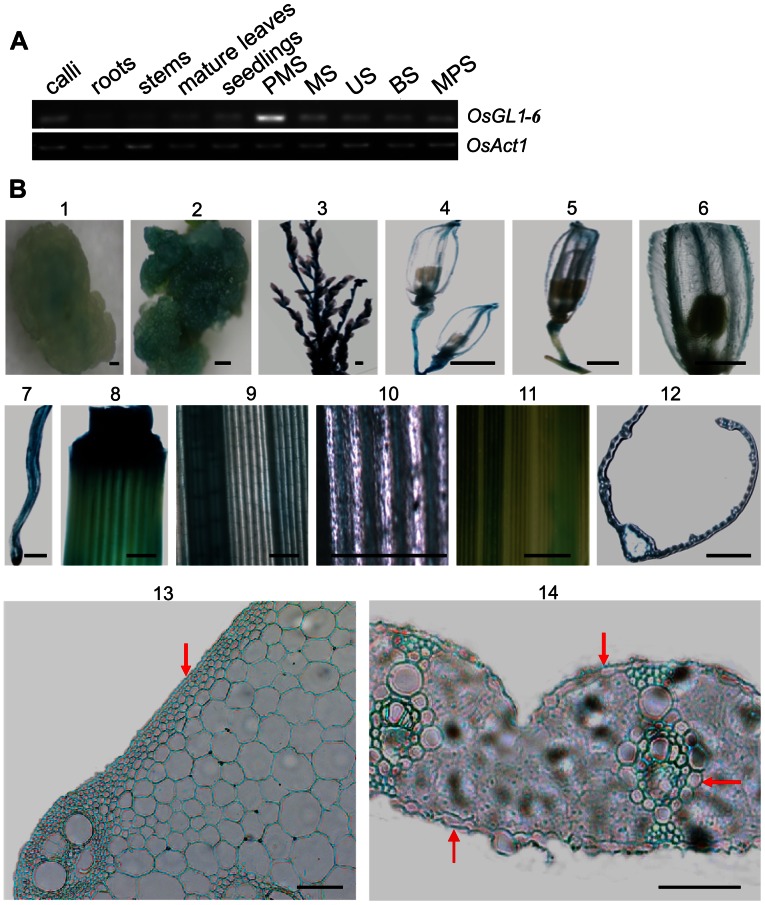
Expression profiles of *OsGL1-6* analyzed using reverse transcription polymerase chain reaction and β-glucuronidase (GUS) staining. (A) Analysis of *OsGL1-6* expression at the transcription level. PMS, spikelets at the pollen mother cell stage; MS, spikelets at the meiosis stage; US, spikelets at the uninucleate stage; BS, spikelets at the binucleate stage; MPS, spikelets at the mature pollen stage. (B) GUS staining analysis of the *OsGL1-6*P:*GUS* transgenic plants. 1, subcultured Hgy-resistant calli; 2, differentiated Hgy-resistant calli; 3, spikelets at the meiosis stage; 4, spikelets at the uninucleate stage; 5, spikelets at the binucleate stage; 6, local amplification of spikelets at the binucleate stage; 7, root; 8, stem; 9, young leaf; 10, local amplification of the young leaf; 11, mature leaf; 12, cross-section of the mature leaf; 13, enlarged cross-section of the stem; 14, enlarged cross-section of the mature leaf. The different stages of spikelet development were collected according to Feng et al. [53]. Red arrows indicate the epidermis cell layer and vascular bundle. Scale bars = 1mm in (1) to (12) and 50 µm in (13) to (14).

To investigate the expression pattern of the *OsGL1-6* gene, a semi-quantitative RT-PCR analysis was performed. As shown in Figure 2A, *OsGL1-6* was expressed in all examined tissues, and the highest expression was observed in the spikelets at the pollen mother cell stage. To confirm the RT-PCR results and further determine the precise expression pattern of *OsGL1-6*, transgenic rice lines expressing the *GUS* reporter gene under the control of the *OsGL1-6* promoter were generated. A total of 10 independent transformed plants were obtained, and the heterozygotes and homozygotes of three transgenic lines were used for GUS analysis with Zhonghua11 as a control.

**Figure 3:**
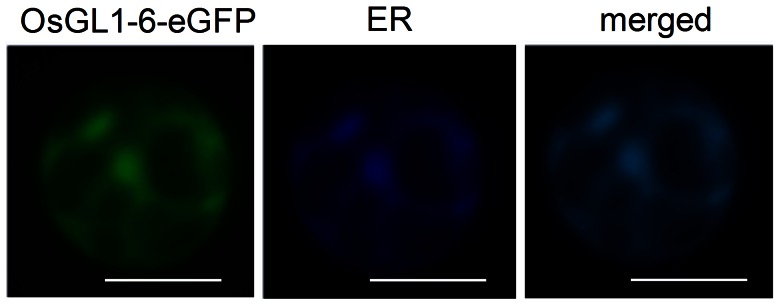
The transient expression of OsGL1-6-eGFP in rice leaf sheath protoplasts. Transient expression vector 35S:*OsGL1-6*-*eGFP* and the transient expression vector *CFP-KDEL* (endoplasmic reticulum [ER] marker) were used to transform the rice protoplasts. Merged, nestification of OsGL1-6-eGFP and the ER marker. Scale bars = 10 µm.

**Figure 4:**
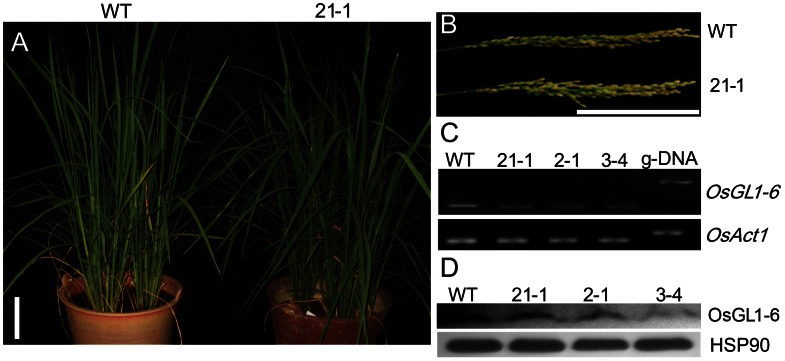
Phenotypic and molecular characterization of the transgenic plants. (A) The shape of the transgenic plants at the booting stage. (B) Seed setting. (C) Semi-quantitative reverse transcription polymerase chain reaction (RT-PCR) of the *OsGL1-6* transcript level in the *OsGL1-6* antisense-RNA transgenic plants compared to the WT plants. *OsGL1-6* transcript levels were examined by RT-PCR using p1 and p2 primers (Figure 1A). *OsAct1* was used as a control. (D) Western blot analysis of the OsGL1-6 in the *OsGL1-6* antisense-RNA transgenic plants compared to that in the WT plants. Heat shock protein 90 was used as the reference protein. WT, wild-type. Scale bars = 10 cm.

**Figure 5:**
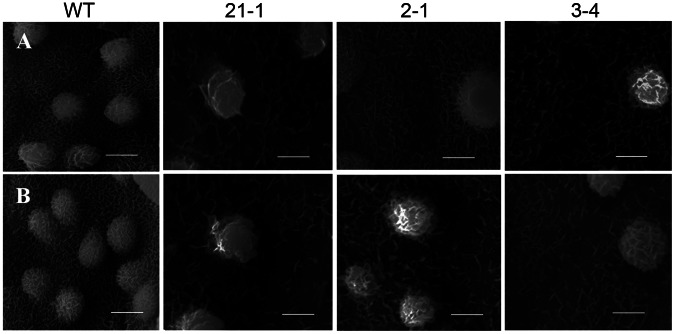
Scanning electron microscopy analysis of the leaf surfaces. (A) Scaning electron microscopy (SEM) analysis of the adaxial leaf surface. (B) SEM analysis of the abaxial leaf surface. The tips of the flag leaves of the wild type and transgenic plants at the booting stage were used for the experiments. Scale bars = 5 µm.

**Figure 6:**
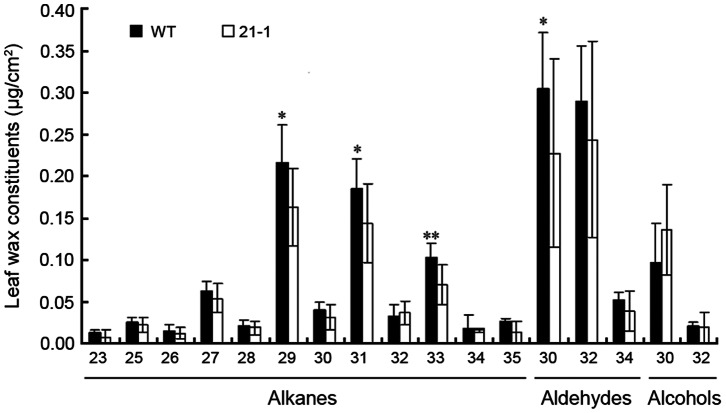
Cuticular wax composition in the leaf of wild type (WT) and ***OsGL1-6*** **antisense-RNA transgenic plants.** Data are shown as mean ± SE and were calculated from three independent experiments. Statistical significance of differences between WT and transgenic plants means is indicated by *(P<0.05) or **(P<0.01).

**Figure 7:**
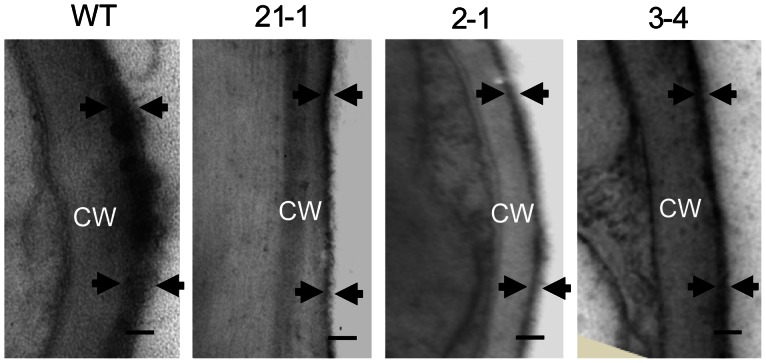
Transmission electron microscopy analysis of leaf cuticle membranes. The tips of the flag leaves of the wild type and transgenic plants at the booting stage were used for the experiments. The zone between the arrows indicates the cuticle. CW, cell wall. Scale bars = 0.2 µm.

**Figure 8:**
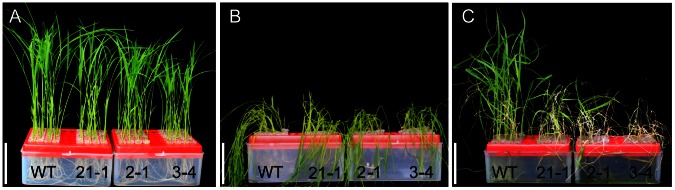
Drought sensitivity experiment between wild type (WT) and *OsGL1-6* antisense-RNA transgenic plants. Seeds from the WT and transgenic plants were germinated on half-strength Murashige and Skoog's medium. The 14-d seedlings were used for the drought treatment. (A) The 14-d seedlings in the air for 0 h. (B) The 14-d seedlings in the air for 10 h. (C) Seedling recovery 7 days after re-watering. Three independent experiments were performed and 24 plants were used for each experiment. Scale bars = 5 cm.

**Figure 9:**
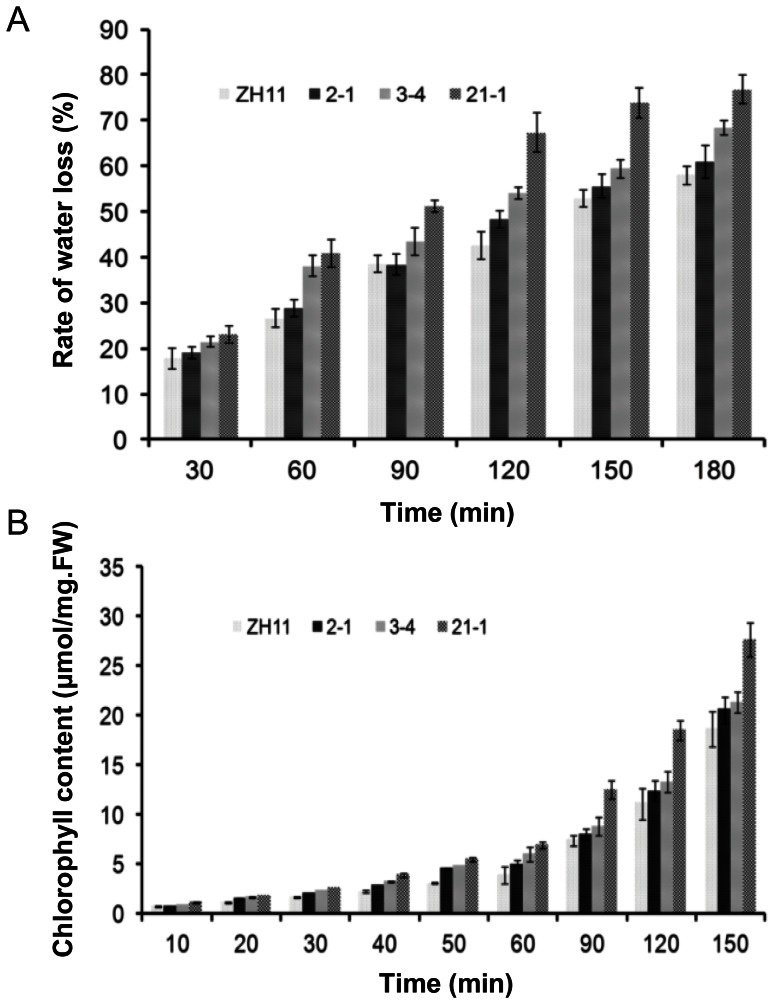
Altered cuticular permeability in *OsGL1-6* antisense-RNA transgenic plants. (A) Water loss rate of the detached leaves of the wild type (WT) and *OsGL1-6* antisense-RNA transgenic plants. The *x* axis shows the different time points, while the *y* axis shows the percentage of free water loss from the leaves. Data are shown as mean ± SE, which were calculated from three independent experiments. (B) Chlorophyll leaching assays using mature leaves of WT and *OsGL1-6* antisense-RNA transgenic plants. Data are shown as mean ± SE, which were calculated from three independent experiments. FW, fresh weight.

Analysis of the GUS activity showed that it was detected in the calli, roots, stems, leaves and spikelets at different developmental stages (Figure 2B). Notably, GUS activity was much weaker in the subcultured calli than in the differentiated calli which feature vascular bundle differentiation (Figures 2B-1, 2B-2). During the meiotic stage, GUS was highly expressed in the node of the tassel, rachis, and pedicel but was not expressed in the glumous flowers (Figure 2B-3). During the uninucleate stage, GUS was expressed in the rachis of the spikelets, pedicel, glumes, and glume veins (Figure 2B-4). During the binucleate stage, GUS staining was detected in the junction of the primary and secondary rachis, pedicels, and glume veins but was not expressed in the rachis of the spikelets (Figure 2B-5, 2B-6). GUS activity remained at high levels in the roots, stems and leaves (Figures 2B-7–14), and its expression was concentrated in the vascular bundle area (Figures 2B-6–14). Strong GUS activity was also detected in the epidermal cells of the stems and leaves (Figures 2B-13 and 2B-14). These results indicate that although the expression of *OsGL1-6* had tissue and developmental stage specificity, most of it was localized in the epidermal cells and vascular bundle area.

### Subcellular Localization of OsGL1-6

Each VLCFA and all of the known wax synthesis-related enzymes are located in the ER [10], [21], [22], [41]. To verify the subcellular localization of OsGL1-6, a 35S:*GL1-6-eGFP* transient expression vector was constructed and co-transfected with *CFP-KDEL* into rice leaf sheath protoplasts. *OsGL1-6* was fused with *eGFP*, which expresses a green fluorescent protein. The ER retention signal *KDEL* was fused with *CFP*, which expresses a deep blue fluorescent protein. Overlap of the GFP and CFP signals results in a light blue fluorescent signal. Fluorescence microscopy revealed that the ER marker fluorescence and the OsGL1-6-eGFP fluorescence overlapped completely, indicating that OsGL1-6 was located in the ER (Figure 3).

### Characterization of *OsGL1-6* Antisense-RNA Transgenic Plants

To investigate the role that *OsGL1-6* plays in wax accumulation, an *OsGL1-6* antisense-RNA vector was constructed and transformed into rice variety Zhonghua11. A total of nine independent transgenic lines were obtained. Phenotype observation showed that most of the transgenic plants exhibited drooping leaves at the booting stage (Figure 4A). However, the pollen fertility (Figure S1) and the seed setting (Figure 4B) did not change compared with those in WT plants. Three independent transgenic lines, i.e., 2-1, 3–4, and 21-1, each of which has a single insertion, were used in the further studies.

Semi-quantitative RT-PCR and Western blot results showed that the *OsGL1-6* expression was significantly decreased in all three transgenic lines (Figures 4C, 4D). We also detected the relative expression levels of *Wda1*, *OsGL1-1* (*WSL2*), and *OsGL1-2* in the transgenic plants, and all three genes showed steady expression compared with those of the WT plants (Figure S2), implying that the expression of three homologous genes was not affected in the transgenic plants.

### Altered Cuticular Wax in *OsGL1-6* Antisense-RNA Transgenic Plants

To investigate the effect of *OsGL1-6* on wax accumulation, scanning electron microscopy (SEM) was used to perform a detailed observation of the leaf surfaces of the WT and *OsGL1-6* antisense-RNA transgenic plants. In the WT plants, the adaxial and abaxial leaf surfaces were both covered with a dense layer of wax crystals, whereas fewer wax crystals were observed on both leaf surfaces in the three transgenic lines (Figures 5A, 5B, and S3). These results suggest that the reduced *OsGL1-6* expression affected the wax accumulation on the leaf surfaces of the transgenic plants.

To more accurately determine the differences in the wax accumulation of the leaf surfaces between the WT and transgenic plants, the leaf cuticular wax was extracted using hot chloroform, and the total wax content was then determined by analytical balancing. The treated leaves were subsequently analyzed using SEM to examine the degree of cuticular extraction, and the results showed that the leaf cuticular wax layer was nearly invisible (Figure S4), indicating that the extraction was thorough. Compared to the WT, the leaf wax contents of the three *OsGL1-6* antisense-RNA transgenic lines were reduced by 36–58% (Table 1), indicating that the decreased expression of *OsGL1-6* in the transgenic plants directly affected the leaf wax accumulation.

**Table 1:** The total leaf cuticular wax content of wild type (WT) and *OsGL1-6* antisense-RNA transgenic plants.

Lines	Wax content(mg/g)	Reduction of the waxcontent (%)
WT	3.31±0.09	
21-1	1.39±0.24**	58
3-4	1.9±0.1**	42.6
2-1	2.12±0.08**	36

Values are given as mean ± SD with three replicates. Significantly different values are represented by asterisks (**) and the mean values are significantly different at *P*<0.01 by the *t-*test between the WT and *OsGL1-6* antisense-RNA transgenic plants.

To determine the exact change in leaf wax accumulation between the WT and *OsGL1-6* antisense RNA transgenic plants, GC-MS was used to analyze the leaf wax composition both qualitatively and quantitatively. The total cuticular wax contents were reduced by 18% in line 21-1 (Table S2). Compared to the WT plants, the alkane and aldehyde contents in the transgenic plants were decreased by 23% and 21%, respectively. However, the primary alcohol content was increased by 30%, and C30 in particular was increased by 41.2% (Figure 6; Table S2), which suggests that *OsGL1-6* is associated with the decarbonylation pathways in wax biosynthesis.

### Altered Cuticle Structure in *OsGL1-6* Antisense-RNA Transgenic Plants

To investigate the effect of *OsGL1-6* on cuticle structure, transmission electron microscopy was used to observe the ultrastructural changes of cross-sections of the WT and transgenic plant leaves. The results showed that cuticle membrane thickness was obviously reduced in the *OsGL1-6* antisense-RNA transgenic plants (30–40 nm), compared to the WT plants (80–100 nm) (Figure 7). The cuticular layers on the WT plants were thicker than those on the transgenic plants. However, unlike the compact or indistinct cuticle proper seen in the WT plants, the cuticle proper of the *OsGL1-6* antisense-RNA transgenic plants showed a fluffy appearance and disorganized bulges (Figure 7), suggesting that the reduced *OsGL1-6* expression in the transgenic plants affected the cuticle structures.

### Altered Drought Sensitivity and Cuticular Permeability in *OsGL1-6* Antisense-RNA Transgenic Plants

Studies have shown that changes in wax accumulation generally lead to changes in plant drought sensitivity and cuticular permeability. As such, drought-sensitivity, water loss, and chlorophyll leaching assays were conducted in both WT and transgenic plants. When the plants were drought stressed, only 4, 4, and 3 of 24 seedlings of the transgenic lines 21-1, 2-1, and 3–4 recovered, respectively, whereas 23 of 24 WT seedlings survived (Figure 8). The drought resistance capacity was also evaluated by measuring water loss from the detached leaves. Compared to the WT plants, the detached leaves from the *OsGL1-6* antisense-RNA transgenic plants lost water more rapidly at all examined time points (Figure 9A). We also checked the leaf cuticular permeability using the chlorophyll leaching method. As shown in Figure 9B, the chlorophyll leached more quickly from the *OsGL1-6* antisense-RNA transgenic plant leaves than from the WT plant leaves. These results indicate that the *OsGL1-6* antisense-RNA transgenic plants are more sensitive to drought stress than the WT plants, implying that the *OsGL1-6* gene may be involved in rice drought protection.

## Discussion

In this study, we constructed an *OsGL1-6* antisense transgene driven by its cognate promoters and obtained several independent transgenic plants with decreased *OsGL1-6* expression. The *OsGL1-6* antisense-RNA transgenic rice plants showed drooping leaves at the booting stage as well as a smooth leaf surface, decreased leaf cuticular wax deposition and cuticle thickness, and enhanced drought sensitivity. *OsGL1-6* shared high similarity to *CER1*, *WAX2* in *Arabidopsis* and *Wda1, OsGL1-1*(*WSL2*), and *OsGL1-2 *in rice. Our study showed that the *OsGL1-6* antisense-RNA transgenic plants exhibited reduced amounts of leaf total cuticular wax, alkane, and aldehyde but increased amounts of primary alcohol, especially C30, indicating that *OsGL1-6* was primarily associated with leaf cuticular wax accumulation. However, the *cer1* mutants exhibited a significant reduction in alkanes, secondary alcohols, and ketones as well as an increased aldehyde content [54].

*CER1* encodes an aldehyde decarboxylase and functions in both stem wax and pollen development [31]–[34]. The leaves and stems of the *wax2* mutants showed proportional deficiencies in aldehyde, alkane, secondary alcohol, and ketone contents as well as increased primary alcohol contents, especially the C30 primary alcohols on the stem surface [35]. Compared with the WT plants, the fatty acid, alkane, aldehyde, and primary alcohol contents in the *wda1*, *wsl2*, and *osgl1-2* mutants were all reduced [39], [41], [42]. *Wda1* was expressed in the epidermis cells of anthers and involved in microspore exine development in the tapetum [39]. *OsGL1-1* (*WSL2*) and *OsGL1-2* were involved in leaf wax accumulation and drought resistance [40]–[42]. The different phenotype and effect on wax composition caused by the disruption of *OsGL1-6*, *CER1*, *WAX2*, *Wda1*, *OsGL1-1* (*WSL2*), and *OsGL1-2* function reminded us that they played at least partially distinct roles in plant growth and development as well as in the biosynthesis of cuticular wax, although these genes shared high sequence similarity (identity >30%).

Studies have shown that mutations of wax biosynthesis-related genes affect the cuticular wax accumulation or cuticle structure. For example, *CER1*, *CER6*, *GL8*, and *WSL1* affect cuticular wax accumulation [12], [14], [16], [31], [55], while genes, such as *LACS2* affect cuticle structure [56]. However, *WAX2*, *GL1*, *Wda1*, *OsGL1-1* (*WSL2*), *OsGL1-2, WXP1*, and *WIN1* affect both cuticular wax accumulation and cuticle structure [27]–[29], [35], [38]–[42]. In this study, leaf cuticular wax deposition in the *OsGL1-6* antisense-RNA transgenic plants was significantly reduced, while the wax contents and cuticle thickness were also reduced compared with those in the WT plants (Figures 5–7 and Table 1). These results suggest that *OsGL1-6* is involved in leaf cuticular wax accumulation and cuticle membrane formation, a finding that is similar to those of its homologous genes *Wda1*, *OsGL1-1* (*WSL2*), and *OsGL1-2* in rice.

Many wax biosynthesis-related genes – such as *CER6*, *CER4*, *WAX2, MAH1*, *Wda1*, and *OsGL1-1*– have been reported to be highly expressed in epidermal cells [12], [20], [22], [36], [39], [40]. GUS staining analysis showed that *OsGL1-6* was also highly expressed in epidermal cells (Figures 2B-13, 2B-14), indicating that *OsGL1-6* may be involved in wax biosynthesis. Although GUS staining was also observed in the calli, roots, stems, and different floral organs during different developmental stages (Figure 2B), the reduced expression of *OsGL1-6* caused phenotypic alterations in the leaves but not in the other organs in which it is expressed. *OsGL1-6* homologs in the rice genome might compensate for the depletion of the *OsGL1-6* gene in these organs.

In addition, GUS activity was stronger in the differentiated calli which have vascular bundle differentiation than that in the subcultured calli (Figure 2B-1, 2B-2). The expressions of *OsGL1-6* in the roots, stems, and leaves were also concentrated within the vascular bundle regions (Figures 2B-6, 2B-7, 2B-12–14). Some genes involved in wax biosynthesis are reported to be highly expressed in the vascular bundles [21], [40], [57]. However, it remains unknown why this is the case. Early studies in maize and barley reported that the *nsLTPs* gene was expressed in the vascular bundles [58], [59]. The transcripts of several *nsLTPs* in common wheat were also observed in the vascular bundles of leaves, roots, and florets of transgenic rice plants detected by the *GUS* reporter gene [60]. Although *in vitro* experiments have shown that non-specific lipid transfer proteins have the capacity to transfer lipids between lipid bilayers [61], their exact biological function remains unclear, especially the roles that they play within the vascular tissues [60]. Our study showed that *OsGL1-6* was highly expressed in the vascular bundles, a finding that implies that *OsGL1-6* may have functions in addition to contributing to leaf wax accumulation.

One of the important functions of cuticular wax is to prevent non-stomatal water loss from the aerial parts of terrestrial plants [62]; thus, it is closely correlated to plant drought resistance. In our study, the *OsGL1-6* antisense-RNA transgenic plants showed increased chlorophyll leaching and water loss rates as well as enhanced drought sensitivity. The wax gene mutants generally exhibited reduced wax accumulation, increased chlorophyll leaching and water loss rates, and enhanced drought sensitivity [35], [42], [63], whereas the overexpression of wax biosynthesis-related genes generally resulted in increased wax accumulation and enhanced plant drought tolerance [28], [29], [33], [42]. The drought susceptibility of the *OsGL1-6* antisense-RNA transgenic plants was in agreement with their deficient cuticles and positively correlated with the reduced accumulation of the leaf cuticular wax, implying its role in drought stress resistance.

This study reported that *OsGL1-6* is involved in both leaf cuticular wax accumulation and drought resistance. Thus, genetic modification of *OsGL1-6* may have great potential for improving the drought resistance of rice. In addition, the availability of these *OsGL1-6* antisense-RNA transgenic plants will be convenient for further studies of the role of waxes in response to other types of environmental stress.

## Supporting Information

Figure S1 **Pollen fertility observation of wild type (WT) and** ***OsGL1-6*** **antisense-RNA transgenic plants.** (A) WT; (B) *OsGL1-6* antisense-RNA transgenic rice plants. The spikelets were collected prior to flowering on the flowering day and fixed in formalin-acetic acid-alcohol fixative. The anthers of same spikelet were pressed on a glass slide and stained with potassium iodide (1% I_2_-KI). The pollen morphology and staining reactions were observed under microscope. Scale bars = 100 µm.
(TIF)

Figure S2 **Relative expression of the three homologous genes of** ***OsGL1-6*** **associated with wax synthesis in the wild type and** ***OsGL1-6*** **antisense-RNA transgenic plants.**
(TIF)

Figure S3 **Scanning electron microscopy analysis of the leaf surfaces.** (A) Scanning electron microscopy (SEM) analysis of the adaxial leaf surface. (B) SEM analysis of the abaxial leaf surface. Scale bars = 5 µm.
(TIF)

Figure S4 **Scanning electron microscopy (SEM) analysis of the leaves after hot chloroform extraction.** (A) SEM analysis of the adaxial leaf surface before the hot chloroform extraction; (B) SEM analysis of the abaxial leaf surface before the hot chloroform extraction; (C) SEM analysis of the adaxial leaf surface after the hot chloroform extraction; (D) SEM analysis of the abaxial leaf surface after the hot chloroform extraction. Scale bars = 5 µm.
(TIF)

Table S1 **Sequences of the primers used in this study.**
(DOC)

Table S2 **Detailed wax contents in the leaves of WT and** ***OsGL1-6*** **antisense-RNA transgenic plants.**
(DOC)
