# Supplementary figures and images for: Rice OsGL1-6 Is Involved in Leaf Cuticular Wax Accumulation and Drought Resistance
Source: PLoS One. 2013 May 31;8(5):e65139. doi: 10.1371/journal.pone.0065139 (PMC3669293; doi:10.1371/journal.pone.0065139)

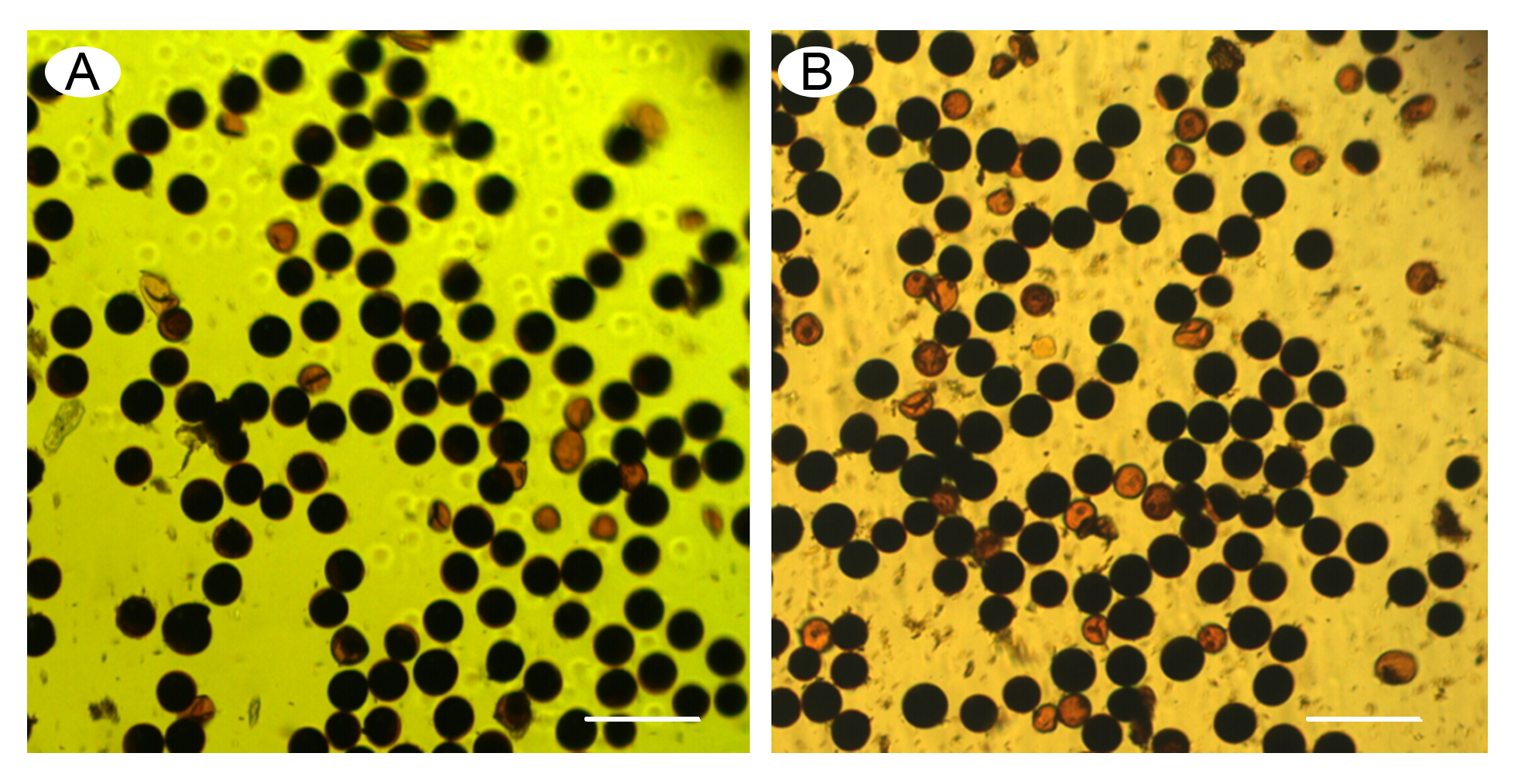

Supplement: Figure S1 — Pollen fertility observation of wild type (WT) and OsGL1-6 antisense-RNA transgenic plants. (A) WT; (B) OsGL1-6 antisense-RNA transgenic rice plants. The spikelets were collected prior to flowering on the flowering day and fixed in formalin-acetic acid-alcohol fixative. The anthers of same spikelet were pressed on a glass slide and stained with potassium iodide (1% I2-KI). The pollen morphology and staining reactions were observed under microscope. Scale bars = 100 µm. (TIF) [file pone.0065139.s001.tif]

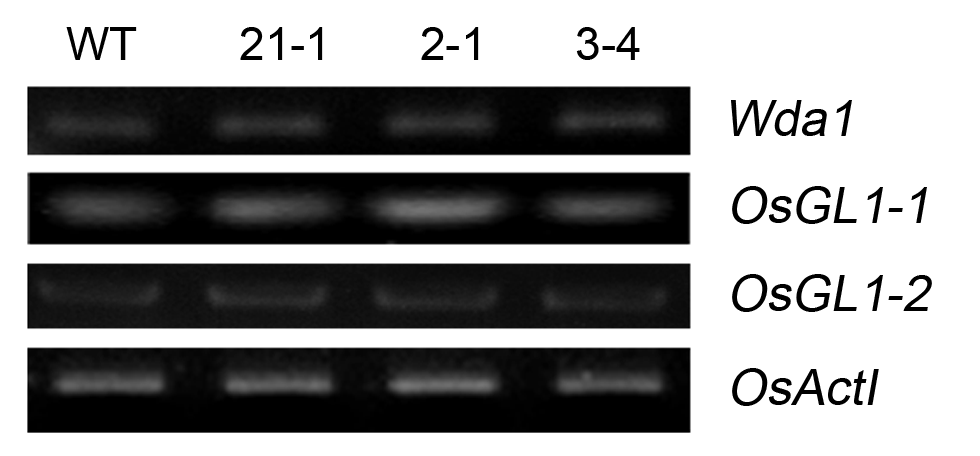

Supplement: Figure S2 — Relative expression of the three homologous genes of OsGL1-6 associated with wax synthesis in the wild type and OsGL1-6 antisense-RNA transgenic plants. (TIF) [file pone.0065139.s002.tif]

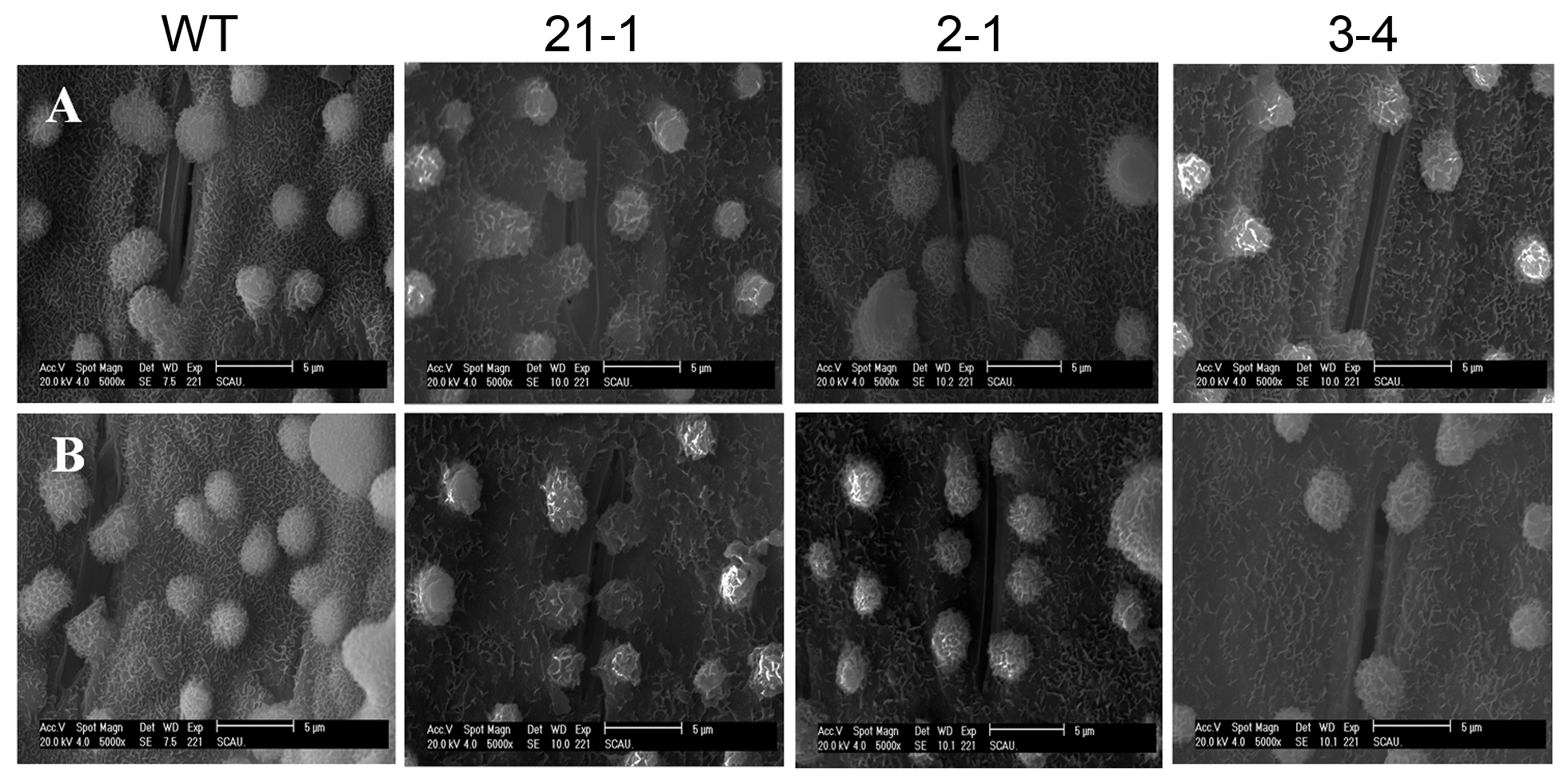

Supplement: Figure S3 — Scanning electron microscopy analysis of the leaf surfaces. (A) Scanning electron microscopy (SEM) analysis of the adaxial leaf surface. (B) SEM analysis of the abaxial leaf surface. Scale bars = 5 µm. (TIF) [file pone.0065139.s003.tif]

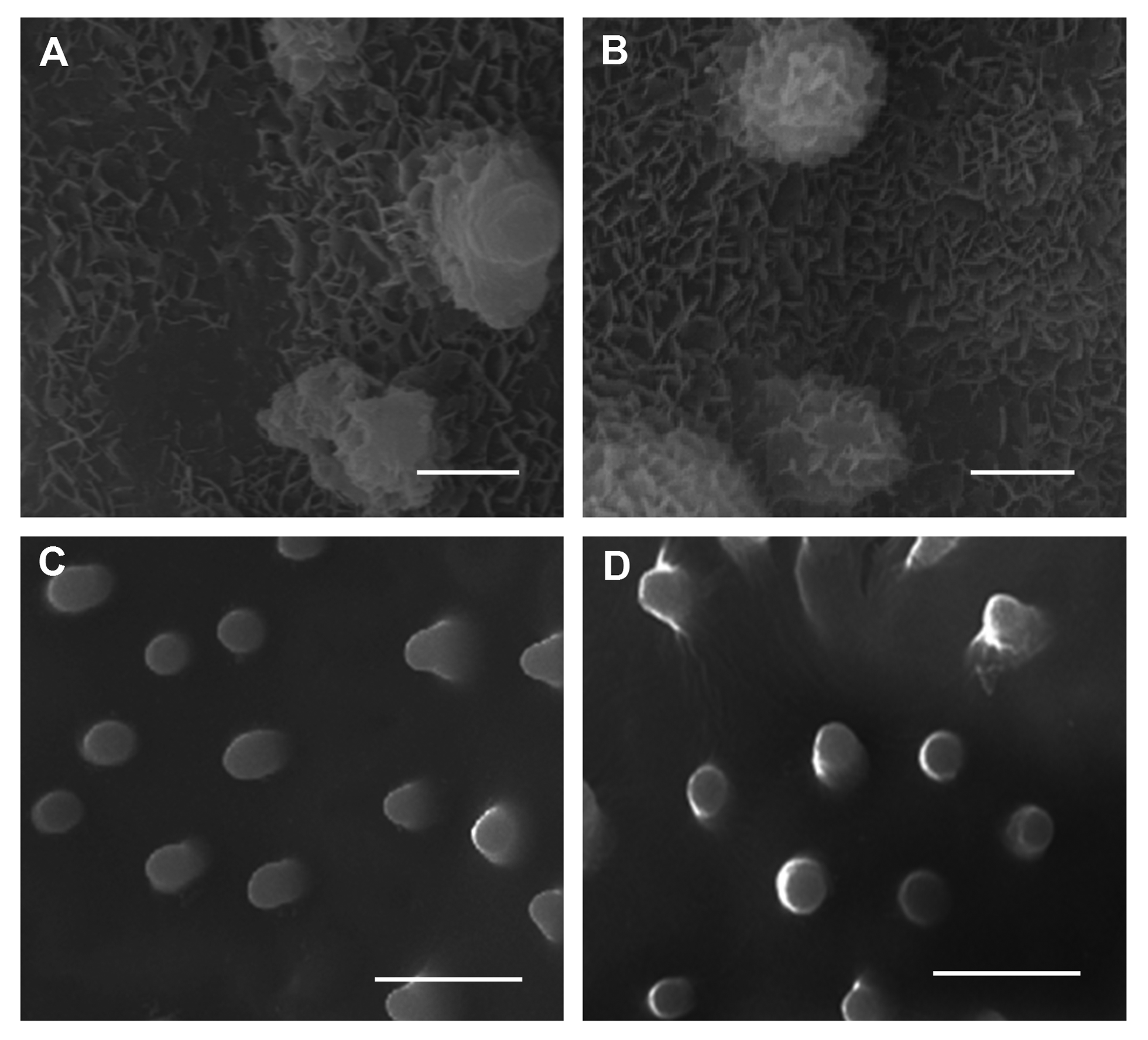

Supplement: Figure S4 — Scanning electron microscopy (SEM) analysis of the leaves after hot chloroform extraction. (A) SEM analysis of the adaxial leaf surface before the hot chloroform extraction; (B) SEM analysis of the abaxial leaf surface before the hot chloroform extraction; (C) SEM analysis of the adaxial leaf surface after the hot chloroform extraction; (D) SEM analysis of the abaxial leaf surface after the hot chloroform extraction. Scale bars = 5 µm. (TIF) [file pone.0065139.s004.tif]
