# Supplementary material for: Rice OsGL1-6 Is Involved in Leaf Cuticular Wax Accumulation and Drought Resistance
Source: PLoS One. 2013 May 31;8(5):e65139. doi: 10.1371/journal.pone.0065139 (PMC3669293; doi:10.1371/journal.pone.0065139)
Supplement: Table S1 — Sequences of the primers used in this study. (DOC) [file pone.0065139.s005.doc]

**Table S1. Sequences of the primers used in this study**

| Name of primers | Sequence (5' to 3') | Description |
| --- | --- | --- |
| *OsGL1-6*Pf | AAAAGGATCCGGTTTGGAGATACAATCTGTG | *Bam*H I |
| *OsGL1-6*Pr | AAAAGTCGACTAGAGGCCATGATAGCTAGC | *Sal* I |
| *OsGL1-6*Af | AAAAAAGCTTACAAGTCAAGCGACGAGCTG | *Hin*d III |
| *OsGL1-6*Ar | AAAAGTCGACTTACCATCCTCACCTGGATC | *Sal* I |
| *OsGL1-6*Gf | AAAAAAGCTTGGTTTGGAGATACAATCTGTG | *Hin*d III |
| *OsGL1-6*Gr | AAAAGGATCCTAGAGGCCATGATAGCTAGC | *Bam*H I |
| *OsGL1-6*-eGFPf | AAAAGAATTCATGGCCTCTAAGCCAGGGCC | *Eco*R I |
| *OsGL1-6*-eGFPr | AAAAAAGCTTACAGCCCGCCACGGCCATCA | *Hin*d III |
| *CFP-KDEL*-1 | AACCGTCGACATGGTG AACAAACACTTCTT | *Sal* I |
| *CFP-KDEL*-2 | CCTCGCCCTTGCTCACCATCTCCCAGTTGAAT  CCCTGAA |  |
| *CFP-KDEL*-3 | TTCAGGGATTCAACTGGGAGATGGTGAGCA  AGGGCGAGGA |  |
| *CFP-KDEL*-4 | AAAAGCGGCCGCTTAGAGCTCATCTTTCTTG  TACAGCTCGTCCA | *Not* I |
| *OsGL1-6*-pETf | AAAAGAATTCTCACGCACCTCACCACGC | *Eco*R I |
| *OsGL1-6*-pETr | AAAAAAGCTTCCCGCCACGGCCATCATG | *Hin*d III |
| *OsGL1-6*-RTf | GTCATGCAGTTACAGCAGCA |  |
| *OsGL1-6*-RTr | TCGTCGTATGGCCGGAATC |  |
| *Wda1*-RTf | CTGCCAAGGAGGGTTATGAG |  |
| *Wda1*-RTr | CGTAATTCCGTATGCTGGA |  |
| *OsGL1-1*-RTf | GTTCGTCTCGTCGATCCAAC |  |
| *OsGL1-1*-RTr | CTCATCTCTTTATGTATCCAAC |  |
| *OsGL1-2*-RTf | GCACAAAACTGCAAGACGTG |  |
| *OsGL1-2*-RTr | CACCAAATCCCACAAGATCG |  |
| *OsAct1*f | CGTCTGCGATAATGGAACTG |  |
| *OsAct1*r | TCTGGGTCATCTTCTCACGA |  |
