# Supplementary material for: Rice OsGL1-6 Is Involved in Leaf Cuticular Wax Accumulation and Drought Resistance
Source: PLoS One. 2013 May 31;8(5):e65139. doi: 10.1371/journal.pone.0065139 (PMC3669293; doi:10.1371/journal.pone.0065139)
Supplement: Table S2 — Detailed wax contents in the leaves of WT and OsGL1-6 antisense-RNA transgenic plants. (DOC) [file pone.0065139.s006.doc]

**Table S2.** Detailed wax contents in the leaves of WT and *OsGL1-6* antisense-RNA transgenic plants

|  | Number^a^ | WT | 21-1 |
| --- | --- | --- | --- |
|  |  | Mean ±SD^b^ µg/cm^2^ (%) | Mean ±SD^b^ µg/ cm^2^ (%) |
| Alkane | C23 | 0.013±0.004 (0.83) | 0.008±0.008 (0.63) |
|  | C25 | 0.026±0.005 (1.70) | 0.022±0.009 (1.77) |
|  | C26 | 0.016±0.006 (1.02) | 0.013±0.007 (1.00) |
|  | C27 | 0.063±0.012 (4.12) | 0.054±0.017 (4.33) |
|  | C28 | 0.022±0.006 (1.41) | 0.019±0.008 (1.52) |
|  | C29 | 0.216±0.045 (14.15) | 0.162±0.046(12.91) |
|  | C30 | 0.040±0.009 (2.64) | 0.032±0.014(2.53) |
|  | C31 | 0.185±0.036 (12.13) | 0.144±0.046(11.44) |
|  | C32 | 0.033±0.014 (2.13) | 0.037±0.014 (2.92) |
|  | C33 | 0.102±0.017 (6.71) | 0.070±0.023 (5.59) |
|  | C34 | 0.019±0.016 (1.21) | 0.015±0.002(1.26) |
|  | C35 | 0.028±0.003 (1.80) | 0.014±0.013(1.08) |
| Total Alkane |  | 0.762±0.113(49.87) | 0.590±0.230(46.98) |
| Aldehyde | C30 | 0.304±0.066 (19.94) | 0.227±0.113 (18.13) |
|  | C32 | 0.290±0.064(19.00) | 0.244±0.117(19.41) |
|  | C34 | 0.053±0.009 (3.43) | 0.039±0.024(3.11) |
| Total Aldehyde |  | 0.647±0.140(42.38) | 0.510±0.254(40.65) |
| 1-alcohol | C30 | 0.097±0.046(6.35) | 0.136±0.053 (10.86) |
|  | C32 | 0.022±0.003(1.41) | 0.019±0.018 (1.51) |
| Total 1-alcohol |  | 0.119±0.044 (7.75) | 0.155±0.059 (12.37) |
| Total |  | 1.527±0.8204 (100) | 1.255±0.36 (100) |

^a^ indicates length of carbon chains in various very-long-chain fatty acids and their derivatives.

^b^ denotes standard deviation (n=3).
